# Prevalence of human papilloma virus (HPV) genotypes between outpatients males and females referred to seven laboratories in Tehran, Iran

**DOI:** 10.1186/s13027-022-00421-7

**Published:** 2022-03-05

**Authors:** Iman Rezaee Azhar, Mahmood Yaghoobi, Mir Majid Mossalaeie, Abolghasem Kollaee Darabi, Amir Houshang Nejadeh, Mahbobeh Jamshidi, Ali Ahani, Masoud Karkhane Mahmoodi, Leila Ghalichi, Ayda Shabanzadeh, Angila Ataei-Pirkooh, Arezoo Marjani, Azam Khamseh, Motahareh Shafiei, Parastoo Hosseini, Saber Soltani, Milad Zandi, Parsa Ghafari, Amir Aboofazeli, Azam Ghaziasadi, Seyed Mohammad Jazayeri

**Affiliations:** 1 Research Center for Clinical Virology, Tehran University of Medical Sciences, Tehran, Iran; 2 Aramesh Medical Laboratory, Tehran, Iran; 3 Parseh Pathobiology and Genetics Laboratory, Tehran, Iran; 4 Noor Genetic Laboratory, Ahvaz, Iran; 5 Nejadeh Medical Laboratory, Tehran, Iran; 6 Mendel Genetic and Pathobiology Laboratory, Tehran, Iran; 7 Albert Medical Laboratory, Tehran, Iran; 8 Mental Health Research Center, Psychosocial Health Research Institute, Iran University of Medical Science, Tehran, Iran; 9 Department of Virology, School of Medicine, Iran University of Medical Sciences, Tehran, Iran; 10 Department of Bacteriology and Virology, School of Medicine, Shiraz University of Medical Science, Shiraz, Iran

**Keywords:** Human papilloma virus, Sexually-transmitted infections, Cervical cancer

## Abstract

**Background:**

Human papilloma virus (HPV) causes the most common sexually-transmitted infection especially among sexually-active individuals. The aim of study was to characterize the molecular characterization of HPV genotypes between 5176 female and male patients.

**Methods:**

HPV DNA was extracted from genital swabs of the study participants and amplified by Real Time Polymerase Chain Reaction (PCR). Genotyping was performed for 2525 cases using REALQUALITY RQ-Multi HPV Detection Kit for the identification of 14 high risk (HR) and 2 low risk (LR) HPV genotypes. Demographic figures were analyzed in correlation with virological data statistically.

**Results:**

Out of 5176 cases from 7 laboratories, 2727 (53%) were positive for HPV, of which. 2372(87%) women and 355 (13%) men were HPV positive. However, in an intra-gender analysis, positive rate was higher in men (355/637, 55.7%) than in women (2372/4539, 52%; *P* value 0.007). HPV positive patients were younger than negative individuals. Positive rate was higher among age categories 20–40. Genotyping was performed for 2525 cases. Out of 1219 (48%) patients who contained single genotypes, 566 (22%) and 653 (26%) harboured HR and LR genotypes, respectively. In females and males, 1189 (54%) and 117 (37%) contained multiple genotypes. No substantial associations were found between different age categories and HR/LR and multiple genotypes distribution.

**Conclusion:**

The prevalence of HPV infection in both genders was high. However, men had a higher rate of infection. These observations highlighted the necessity for a plan for targeted education to younger population in the society as well as application of infection control measures against HPV infection, especially in terms of general population mass HPV vaccination.

## Introduction

Genital infections with human papillomavirus (HPV) are the most common sexually transmitted infections worldwide which affect as high as 90% of sexually active women, depending on world region, population source and methodology for detection [1–3]. Also, HPV infection is recognized as a major causative agent in the development of cervical cancer which remains the fourth most common cancer among women worldwide with an estimated 569,847 new cases and 311,365 deaths in developing countries in 2018 [4–6]. Currently, more than 150 HPV genotypes have been identified and about 40 are known to transmit through sexual contact and to infect the anogenital region [7–9].

According to Institut Català d’Oncologia and International Agency for Research on Cancer Information Centre on HPV and Cancer (ICO/IARC) report released on 2018, about 917 new cervical cancer cases are diagnosed annually in Iran. Also, cervical cancer ranked as the 16th leading cause of female cancer which accounted as being the 10th most common female cancer in women aged 15–44 years old in Iran [10]. In line with the Iranian published data, the prevalence of HPV in different female cervical specimens has been reported to be: between 5.5 and 9.4% in normal cytology specimens [11–13]; between 61.7 and 65.3% in Cervical Intraepithelial Neoplasia (I–III) samples [12, 14, 15] and between 75.2 and 87% in cervical cancer specimens with a high heterogeneity among studies [16–18]. In females, the prevalence of the different high risk HPV genotypes has been determined and analyzed within the setting of either cytologic examinations and HPV genotyping (as co-testing) or HPV genotyping only (as primary testing) with the aim of screening for and diagnosing precancerous and cancerous lesions worldwide. Therefore, screening of women for precancerous and cancerous lesions by cytologic examinations and HPV typing still of paramount importance.

Iranian published data on HPV especially high risk (HR) genotypes in men are scare. Current data revealed HPV prevalence of 9.5–30% among men referral to diagnostic centers [19, 20], and in penile and anal specimens obtained from male participants, high risk (HR) and low risk (LR) HPV genotypes were 5.5% and 13.7% respectively [21].

The objectives of this survey were: first, to identify the prevalence of HPV DNA positivity and genotypic identification among outpatients female’s cervical secretions under the standard protocols for cervical cancer screening who referred to seven medical laboratories in Tehran Metropolitan between 2017 and 2021 and also in their male partners of positive cases, second, to recognize the HPV prevalence in genital specimens from referral single males and females; and third, to identify demographical characteristics of positive cases.

## Methods

### Clinical specimens

This cross-sectional retrospective investigation was undertaken on different cervical specimen obtained from outpatient females who referred to seven medical laboratories located in Tehran Metropolitan (Noor, Parseh, Aramesh, Mandel, Albert, Nejadeh and Laleh Hospital) collaborative to Research Center for Clinical Virology (RCCV), Tehran University of Medical Sciences between 2019 and 2021. These laboratories are of foremost laboratories which receive many different types of samples from physicians of different specialists including gynecologists, urologists and dermatologists across Tehran province. The participants who were accepted into the survey were asked to complete a written informed questionnaire.

For normal cytology examination, thinpreps (liquid-based cytology) and cervical sections were referred by physicians to the laboratories based on standard cervical cancer screening methodology. However, for those outpatients who were seeking HPV identification outside of normal screening protocols, cervical and vaginal secretions (the latter for virgin females) were obtained both by either physicians or by trained laboratory personnel. For men, genital samples collection were obtained using methodology described by Aguilar et al. [22]. In short, two different swabs were taken from each male genitalia (one for meatus, another one for penile as well as testicular skin and inguinal area). These two specimens were put together in a single collection cryo-tube. Furthermore, a separate urine specimen was taken by advising males to collect their first morning urine in the collection tube. Therefore, two different assays were carried out for men urogenital specimens. Upon delivery, the samples were maintained at – 20 °C until being tested.

The inclusion criteria include females who were asked for HPV detection and typing by their physicians due to abnormal cytological findings and those who were seeking HPV identification regardless of cytological results. For men, those who were requested HPV identification either through their positive sexual partner test results or for those who had recent unsafe sexual activity and were willing to recognize their HPV status. Those abnormal cytology and biopsy specimens which contained precancerous or cancerous lesions were excluded from the study.

### DNA extraction and PCR

Pre-amplification processing of the specimens, DNA extraction, and HPV genotyping were performed at the department of molecular genetics located in each laboratory according to the same protocol provided by quality control supervisors under control by RCCV. HPV DNA was extracted using QIAamp DNA Extraction kit (Qiagen, Hilden, Germany) according to manufacturer’s structure. PCR was performed on the extracted materials using HPV detection and genotyping using REALQUALITY RQ-Multi HPV Detection Kit (AB-Analitica, Italy) which identified 14 HPV high risk as well as 2 low risk HPV-6 and HPV-11 along with other 30 LR genotypes.

### Data collection

All patients’ demographical and virological information were extracted from each laboratory files, then, were transferred to RCCV for data processing and evaluation. All the data were analyzed by two independent trained researches. For genotypic classification, “multiple genotypes” were defined as patients who contained more than one HR HPV genotypes; therefore, no LR genotypes were included in this categorization.

### Statistical analysis

Statistical analysis was performed using SPSS software version 19. Descriptive statistical methods were administered. A chi-square test and a *t*-test were applied to compare categorical and continuous variables between subgroups. *P* values less than 0.05 were considered significant.

## Results

Out of total numbers of 5176 cases from 7 laboratories, 4539 (88%) and 637 (12%) were females and males, respectively (Table 1). Only 10% of cases (538 cases) declared marital status among whom 70% were single (Table 1). No statistical difference was observed between men and women in declaring marital status. Age of the participants ranged from 1 to 72 years with mean (SD) of 33.2 (8.07). More than 84% (3220) of participants were among 20–40 years old age categories (Table 1).

**Table 1:** Demographical and virological characteristics of patient

Characteristics	Total	HPV positive	HPV negative	*P* value
	N = 5176	N = 2727	N = 2300	
Total				
Female	4539 (88%)	2372 (87%)	2053 (89%)	0.007
Male	637 (12%)	355 (13%)	247 (11%)	
Mean age	33.2	32.6	33.8	< 0.001
SD	8.07	7.71	8.33	
Age category				
< 20	89 (2.4%)	57 (2.7%)	32 (2.0%)	< 0.001
21–30	1463 (38.8%)	853 (40.1%)	610 (37.2%)	
31–40	1657 (44.0%)	944 (44.4%)	713 (43.4%)	
41–50	420 (11.1%)	204 (9.6%)	216 (13.2%)	
> 50	139 (3.7%)	69 (3.2%)	70 (4.3%)	
Marital status				
Married	164 (30.5%)	110 (30.6%)	54 (30.2%)	0.911
Single	374 (69.5%)	249 (69.4%)	125 (69.8%)	

Out of total population, 2727 (53%) were positive for HPV, of whom 2372 (87%) and 355 (13%) were females and males, respectively (Table 1). However, in an intra-gender analysis, positive rate was higher in men (355/637, 55.7%) than women (2372/4539, 52%) (*P* value 0.007, Table 1). Mean ages were different between positive and negative cases (32.6 vs. 33.8, respectively, *P* value, < 0.001, Table 1). Therefore, HPV positive patients were younger than negative individuals. Positive rate was higher among age categories 20 to 40, (*P* value < 0.001, Table 1). Marital status was not significantly different between positive and negative subjects (*P* value, 0.911, Table 1). Among who declared their marital status, positive rates were 66.5% and 67% in single and married subjects, respectively, without significant correlations (*P* value 0.911, Table 1).

### Distribution of HPV genotypes

**Fig. 1:**
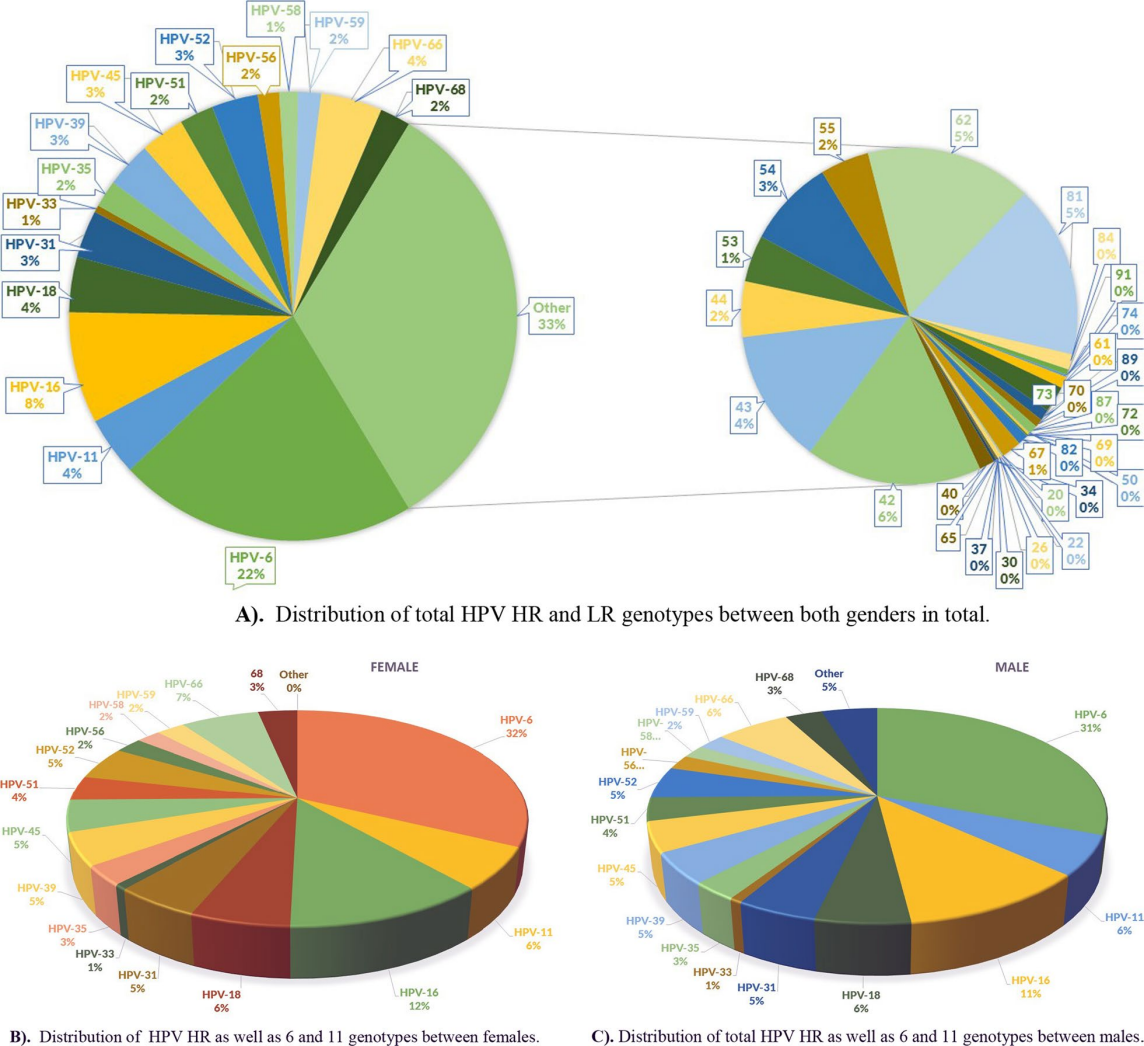
Schematic Distribution of HPV HR and LR genotypes; **A** in both genders, **B** in females and **C** in males. Note: for females and males other LR genotypes than HPV-6 and HPV-11 were skipped for more clarification

Genotyping was performed for 2525 cases (2213 and 312 for females and males, respectively) with finding of total number of 5787 different HPV genotypes. In total, 2396 (41.4%) and 3391 (58.6%) were HR and LR genotypes (Table 2). Females contained 2193 (42.1%) and 3012 (57.9%) HR and LR genotypes, respectively (Table 2). Males contained 203 (34.9%) and 379 (65.1%) HR and LR genotypes, respectively (Table 2). HR genotypes prevalence in the order from highest to lowest contained 16, 66, 18, 31, 39, 45, 52, 68, 51, 35, 56, 58, 59 and 33 (Fig. 1A). HR genotypes in females listed in the order of significance from highest to lowest were: 16, 66, 18, 31, 52, 39, 45, 51, 68, 35, 59, 56, 58 and 33 (Fig. 1B) and in males were: 16, 66, 31, 52, 18, 45, 51, 39, 35, 59, 56, 33, 66 and 58 (Fig. 1C). In both genders, HPV-6 was the most prevalent HPV genotype (32% and 31% in females and males, respectively) (Fig. 1B, C). In terms of intra-gender analysis, HR genotypes 16 and 66 observed in 12% and 7% of females and in 11% and 6% of males, respectively (Fig. 1B, C). HPV-11 was the next most prevalent LR genotype (6% in each gender) (Fig. 1B, C).

**Table 2:** Frequency of HR, LR and other genotypes in females and males

Gender	Total		Female		Male	
Genotypes	N	%	N	%	N	%
Total	5787	100.0	5205	100.0	582	100.0
High risk	2396	41.4	2193	42.1	203	34.9
Total low risk	3391	58.6	3012	57.9	379	65.1
6, 11	1498	25.9	1256	24.1	242	41.6
Other low risk	1893	32.7	1756	33.7	137	23.5

Of total 3391 LR genotypes, 1498 (25.9%) were belonged to HPV genotypes 6 and 11 and 1893 (32.7%) related to other HPV LR genotypes (Table 2). Low risk genotype 6 and 11 was detected in 1256 (24.1%) and 242 (41.6%) of genotypes detected in females and males, respectively (Table 2). Other genotypes were found in 1756 (33.7%) of females and 137 (23.5%) of males, respectively with lower frequencies (Table 2 and Fig. 1A).

**Fig. 2:**
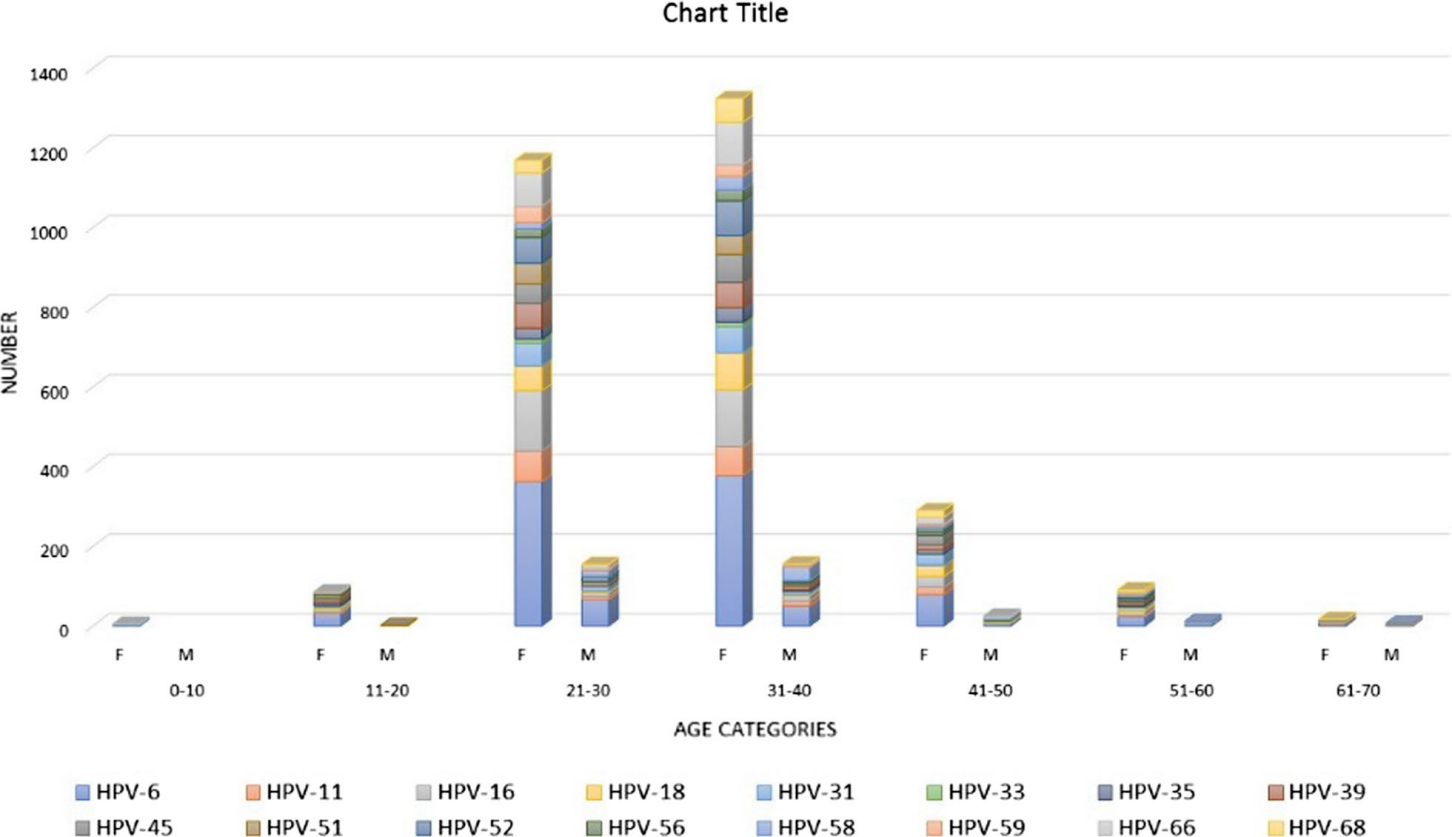
Distribution of total HPV HR as well as 6 and 11 genotypes between genders according to different age categories

The distribution of LR and HR genotypes in both genders between different age categories showed that 31–40 and 21–30 age groups contained the highest prevalence, respectively, followed by 41–50 and 21–30 age groups (Fig. 2). In terms of HPV genotypes risk, of 1219 (48%) patients who contained single genotypes, 566 (22%) and 653 (26%) harboured HR and LR genotypes, respectively; and 1306 (52%) of patients had multiple genotypes (Table 3). In females and males 1189 (54%) and 117 (37%) contained multiple genotypes (*P* value, < 0.001, Table 3). There were no significant correlations between marital status of subjects in terms of HR, LR and multiple genotypes (*P* value, 0.329, Table 3). Also, no substantial associations were found between different age categories and HR/LR and multiple genotypes distribution (*P* value, 0.560, Table 3).

**Table 3:** Comparison of HPV HR and LR single and multiple genotypes distribution according to demographical characteristics

Characteristics	Single genotypes		Multiple genotypes 1306 (52%)	Total 2525	*P* value
	Low risk 653 (26%)	High risk 566 (22%)			
Gender					
Female	507 (23%)	517 (23%)	1189 (54%)	2213	< 0.001
Male	146 (47%)	49 (16%)	117 (37%)	312	
Age category					
< 20	12 (2.4%)	10 (2.3%)	33 (2.9%)	55 (2.7%)	0.560
21–30	208 (42.4)	154 (35.6%)	462 (41.1%)	824 (40.3%)	
31–40	208 (42.4)	206 (47.7%)	494 (43.9%)	908 (44.4%)	
41–50	45 (9.2%)	46 (10.65)	104 (9.2%)	195 (9.5%)	
> 50	17 (3.5%)	16 (3.7%)	32 (2.8%)	65 (3.2%)	
Marital status					
Married	17 (27.9%)	12 (23.1%)	81 (32.9%)	110 (30.6%)	0.329
Single	44 (72.1%)	40 (76.9%)	165 (67.1%)	249 (69.4%)	

## Discussion

Scares efficient data exists for HPV prevalence among Iranian outpatient population. A waste majority of Iranian published data has been undertaken on frozen or stored samples in hospitals and research centers. However, the present investigation focused on fresh samples from referral specimens to well-known laboratories in Tehran Metropolitan. Unpublished data from Iran indicates that since the last decade, genital tract HPV infection has been growing expeditiously among Iranian sexually-active population regardless of age, marital status and economic situation. Two main factors for this significant rising include: the absence of sex education for young society as well as limited access to HPV vaccine in the country. Similarly, the considerable increased incidence of HPV infections in many countries has been accused to be related to an early start of sexual activity and change in sexual behaviors including great numbers of sexual partners, and inadequate preventive measures. Undoubtedly, both factors could have been attributed to Iranian population.

Present study showed that both genders showed a somehow high HPV prevalence; 55.7% and 52% for men and women, respectively. Other national published observations indicated the prevalence of HPV infection in cervical/vaginal secretions between 5.5% and 57.4% of females with different study populations and methodologies [11, 21, 23–27]. Nevertheless, HPV in Iranian males in four studies ranged between 9.5 and 54% [19–21, 28].

Our results showed that in both genders, HPV infection did not differ in different age categories. Nevertheless, HPV positive rates and high-risk genotypes were commonly found in ages between 20 and 40 years old, without significant correlations. Although this figure was similar to other studies for men; however, for women, international published data showed an increase in HPV prevalence in the middle age and afterwards a decline with age [29, 30]. Mobini Kesheh et al., found a high burden of the HPV infection was observed at ranges of 30 and 44 years (51.8%) with a peak at ranges between 30 and 32 years [21]. Sabet et al. found that in both genders, 26–35 years old contained more than half of HPV positive cases [28]. Bitarafan et al. found that 26–46 years old group contained the most frequent HPV positivity [27]. One of the key messages raised form those above Iranian investigations could be the necessity for prompt targeted immunization against HPV for ages between 20 and 40 or even earlier age periods.

Among who declared their marital status, positive rates were 66.5% and 67% in single and married subjects, respectively, without significant correlations. This finding might be related to the fact that in the last two decades, Iranian single male and females (including those who experienced divorce), have been engaged in sexual relationship, especially multi-partnership experiences. We did not find this finding in other above-mentioned Iranian Investigations It mightily because marital status was not included in those studies due to the stigma about this type of relationship in Iranian society.

Interestingly, we observed that in both genders, HPV-6, HPV-11, HPV- 16 and HPV-66 genotypes were the most common HPV genotypes. Two Iranian studies were evaluated the prevalence of HPV genotypes in both genders among population. In the largest Iranian survey on 10,266 samples from 31 Iranian provinces, Mobini Kesheh et al. found 49.5% (n = 5085) were HPV DNA positive [21], among whom, the most common HPV types were HPV-6 (77.7% and 43.3%) and HPV-11 (13.7% and 11.4%), HPV-16 (5.5% and 16.6%) and HPV-52 (3.2% and 9.6%) between male and female subjects, respectively. In the second survey, Sabet et al. investigated the prevalence of HPV genotypes among both genders of three Eastern Iranian provinces [28]. They found a prevalence of positive HPV in 35.3% in the population and the five most common genotypes as being HPV-6 (50%), HPV-11 (10%), HPV-16 (15%), HPV-51 and HPV-53 (the percentages of the two latter genotypes did not specify). In one the largest sample size-studied in Iran undertaken only on women (samples from both inpatients and outpatients), the five most common HR-HPV genotypes were as follows: HPV 16 (16.98%), HPV 52 (8.8%), HPV 18 (7.69%), HPV 39 (7.63%) and HPV 31 (7.45%) [27].

Our survey showed that male had a more positive rate of HPV infection than women, especially in younger ages. Because of the impact of HPV infection on females’ health, there is an influx body of literatures in the database in terms of screening and cervical cancer management for women. However, little is known about HPV infection and its natural history in the male genital tract. There is evidence of steady rising of HPV-related cancers in men [31], especially in men who have sex with men (MSM) and HIV positive men. Furthermore, men could have transmitted HPV infection to women where may lead to cervical cancer and other morbidities [32–36]. In addition, we showed that the prevalence of HR and LR genotypes among male and females were different; HR genotypes were 42.1% and 34.9% whereas LR types were 57.9% and 65.1% in females and males, respectively. These findings were similar to previous finding shown that women had a higher probability of obtaining HR genotypes [37]. Likewise, current investigation showed that multiple genotypes were more common in women than in males (*P* value, < 0.001). Moreover, Pista et al. indicated that multiple HPV infections were more common in younger women, which was in agreement with our results [38]. On the other hand, others illustrated that multiple HPV infections were common in men than in women [39, 40]. This latter finding might have been correlated with the presence of high number of sexual partners during their men sexual life [41].

Present study showed a somehow high prevalence of “other LR genotypes” in genital specimens from both genders (32.7% of total genotypes and 58.6% of LR genotypes). These LR genotypes excluding 6 and 11, have been observed in women with a wide range of frequency between 3.4 and 30.5% [10, 42]. Two Iranian studies found that between 14.36% and 65.3% of Iranian male and female contained other LR genotypes than HPV-6 and HPV-11 [21, 28]. All these “other types” are belonged to Alpha Papillomaviruse, subspecies 1 to 14, which could be found in cervical secretions and genital lesions of both genders as well as in other non-genital lesions such as Epidermodysplasia verruciformis, common warts and flat warts with different frequencies [43, 44]. Although standard operating procedures and test performance were similar between the affiliated laboratories, however, sampling and sample handling together with bias in random sampling among population-studied could not have been ruled out for those above heterogeneities. These discrepancies between results should be investigated in more depth in other surveys with larger sample size in Iran.

Admittedly, there are some limitations and biases for this investigation. First, random sampling between different laboratories could have lessen drawing a definite conclusion. Second, the mere detection of HPV DNA in a specimen does not reinforce an established infection. Third, the cross-sectional nature of study prevents identifying the evolution of HPV infection among population-studied and their following up compared with well-designed cohort studies. Fourth, the above-mentioned HPV prevalence among outpatients could not have mirrored the real HPV epidemiology among general population. According to recent studies on the clinical progression of cervical lesions in women and the importance of this issue, there is a need for deeper and more complete studies in the future that could be the starting point for such studies in the future in the Iranian female population [45–47].

In conclusion, the current study clearly showed that the prevalence of HPV infection in both genders was high. Typically, women had a higher HPV prevalence in the same age category than men. Also, men had a higher rate of infection in younger ages without steady infection patterns in age categories. These observations highlighted the necessity for a plan for targeted education to younger population in the society as well as application of infection control measures against HPV infection, especially in terms of general population mass vaccination.

## Data Availability

All data analysed during this study are included in this article.
